# Parent-adolescent agreement in reported moderate-to-vigorous intensity physical activity during the COVID-19 pandemic

**DOI:** 10.1186/s12889-022-12530-4

**Published:** 2022-02-16

**Authors:** Jason M. Nagata, Catherine A. Cortez, Puja Iyer, Erin E. Dooley, Kyle T. Ganson, Amy A. Conroy, Kelley Pettee Gabriel

**Affiliations:** 1 Department of Pediatrics, University of California, San Francisco, 550 16th Street, 4th Floor, Box 0110, San Francisco, California 94158 USA; 2 Fielding School of Public Health, University of California, Los Angeles, Los Angeles, California USA; 3 Department of Epidemiology, University of Alabama at Birmingham, Birmingham, AL USA; 4 Factor-Inwentash Faculty of Social Work, University of Toronto, Toronto, Ontario Canada; 5 Division of Prevention Science, Department of Medicine, University of California, San Francisco, San Francisco, California USA

**Keywords:** Adolescents, Parents, Physical activity, Moderate-to-vigorous intensity physical activity, Physical activity measurement, COVID-19

## Abstract

**Purpose:**

To describe the agreement between parent- and adolescent- reports of adolescent moderate-to-vigorous intensity physical activity (MVPA) and to determine sociodemographic factors associated with MVPA reporting differences during the COVID-19 pandemic.

**Methods:**

We analyzed data collected in May 2020 from the Adolescent Brain Cognitive Development Study (ABCD, *N* = 4841), a U.S. prospective cohort study. We quantified past weekly adolescent MVPA levels as reported by the parent and adolescent (referent). Intra-class correlation coefficients (ICC) and Bland-Altman plots were used to examine the degree of agreement between parent- and adolescent- reports.

**Results:**

When quantifying adolescent MVPA during the same recall period, median (p25, p75) MVPA (h∙wk.^− 1^) was 2.17 (0.00, 6.00) as reported by adolescents and 1.52 (0.29, 4.75) by parents with a mean difference of 4.89. Statistically significant differences in reports of MVPA were found in households with income > $75,000: on average, adolescents reported higher MVPA levels than their parents. Bland-Altman plots illustrated that, among adolescents reporting no or little MVPA, there was higher parent-adolescent agreement. However, among adolescents reporting high levels of MVPA, there was less agreement between the parent- and adolescent- reports.

**Conclusions:**

Despite more time spent together at home during the pandemic, there was generally low agreement between parent- and adolescent- reports of adolescent MVPA. Future research could examine parent-adolescent agreement of MVPA within the context of device-based measures (e.g., accelerometers), determine reasons for differences in parent-adolescent reporting of MVPA, and inform interventions for improved parental involvement and monitoring of MVPA.

**Supplementary Information:**

The online version contains supplementary material available at 10.1186/s12889-022-12530-4.

## Introduction

Among children and adolescents, moderate-to-vigorous intensity physical activity (MVPA; e.g., running, swimming, or bicycling) is associated with a myriad of health benefits, including lower risk of obesity, higher bone mass, and improved cardiometabolic health [1, 2]. Given the known benefits to child and adolescent health, the U.S. Department of Health and Human Services recommends that school-aged youth 6–17 years of age participate in at least 60 min of MVPA every day [3].

Population-based studies with youth often use self-report-based methods to assess MVPA and estimate the prevalence of those meeting physical activity guidelines. Report-based MVPA measures are generally simple to administer and inexpensive, and thus are commonly used [4, 5]. However, report-based MVPA measures are reliant on memories that are prone to fallicies in retrieval due to decay over time or interference of other memories. Memories may also be filtered by perceptions and biases. Specifically, participant-reported MVPA may be affected by recall [6] and social desirability bias: youth often overestimate their intensity and time spent on MVPA [7]. Use of a proxy respondent varies based on the age of the child with research suggesting that children aged 10 years and older are developmentally mature enough to provide reliable and valid estimates of MVPA [4, 8–10]. To obtain physical activity estimates in younger children, reports from a parent or adult (e.g., teacher) have been used to estimate the child’s MVPA. Prior studies have reported on the agreement and correlation between parent and child accounts of children’s physical activity levels; however, a majority were in children under 10 years of age [4, 8, 10–12]. Notably, levels of agreement between parent- versus child- reports of physical activity may vary between different populations dependent on the child’s age group. To obtain physical activity estimates in younger children, reports from a parent or adult (e.g., teacher) are relied upon to estimate the child’s MVPA. The literature has shown that parent report proxies are typically used for younger children’s activity levels [13–16], while older children typically self-report their own physical activity levels [17–25]. Findings regarding agreement between parent- and child- reports of physical activities have ranged from low to slight to fair in pediatric populations up until age 14 [12, 16, 26, 27]. Prior studies have suggested that using a self-report may be more reliable than parental proxy report in children over the age of 10 [27].

The sociodemographic correlates of discrepancies of parent- and youth- reported physical activity identified in the literature include parent gender [16], youth weight status [12, 16], youth age [28], youth gender [12], family cohesion [28], and family socioeconomic status [12]. These differences may also stem from the fact that parental monitoring of youth physical activity is limited during the time that youth spend at school and parents spend at work [29, 30].

With the onset of the COVID-19 pandemic, social distancing, school closures, and changes in access to community parks and recreational facilities have changed the way in which adolescents participate in MVPA and have led to more time spent at home [31, 32]. Although previous studies reported low agreement in MVPA between parents and adolescents [12, 16, 26, 27], the dyadic agreement between parents and adolescents may have improved during the pandemic due to increased time spent at home together as a result of stay-at-home orders, school and business closures, and travel restrictions [31, 32]. Conversely, with the cancellation of regular routines such as sports team practices and training, competitions, and in-person physical education classes [33], there may have been greater difficulty to recall time spent on MVPA given these activities are often more structured than recreational activity. The stay-at-home orders and other changes during the COVID-19 pandemic [32] allowed for a natural experiment to examine the parental proxy measures of adolescent MVPA in a unique time during which adolescents and their parents may have been in closer quarters with one another and there may have been limited sanctioned opportunities for physical activities.

The present study aims to describe the differences and agreement between parental reports of adolescent physical activity and adolescent self-reports of MVPA. We hypothesize that there will be some agreement between parent- and adolescent- reports of adolescent physical activity during the COVID-19 pandemic given the increased number of hours families spend together and due to fewer MVPA opportunities during the COVID-19 pandemic. Second, we aim to determine sociodemographic correlates of the parent-adolescent dyad physical activity reporting differences. We hypothesized that factors such as adolescent sex, race/ethnicity, education, and household income will be associated with differences in parent and adolescent physical activity reports.

## Methods

### Study population

We analyzed data from the Adolescent Brain Cognitive Development (ABCD) Study, a national (U.S.) prospective cohort study of brain development and health among 11,875 adolescents. The University of California, San Diego provided centralized institutional review board (IRB) approval and each participating site received local IRB approval. Written informed consent and assent were obtained from the parent and adolescent, respectively, to participate in the ABCD Study. Analyses included data from the ABCD Study COVID Rapid Response Research (RRR) Survey 1 (sent between May 16–22, 2020). During this time, almost all states had closed or limited operation of gyms and more than half of states closed all nonessential businesses, although policies varied by U.S. state and counties and cities within these states [32]. Participant pairs were excluded from the analysis if they had completed the COVID RRR surveys out of order or had missing data for parent or adolescent sociodemographic characteristics and reported MVPA, resulting in an analytic sample of 4841 participants.

### Measures

#### Sociodemographic characteristics

Parents were asked to respond to the following sociodemographic items in relation to their adolescent: adolescent sex (male or female) and adolescent race/ethnicity (White, Latinx/Hispanic, Black, Asian, Native American, other). Additional characteristics included household income (less than $75,000 and $75,000 and greater, as this approximated the median household income in the U.S.) and highest parent education (high school or less versus college education or more).

#### Moderate-to-vigorous intensity physical activity (MVPA)

Both parents and adolescents reported daily duration (hours and minutes) and frequency (days per week) the adolescent spent participating in MVPA during the past week in the COVID RRR Survey (see Supplemental Appendix). These questions were adapted from the Youth Risk Behavior Survey (YRBS) [34, 35] and the International Physical Activity Questionnaire (IPAQ) Short F form [36, 37] and included prompts such as running, aerobics, or bicycling. Continuous MVPA estimates were computed as the product of reported duration (h·d^− 1^) and frequency (d∙wk.^− 1^) and expressed as h∙wk.^− 1^. Given the age of the adolescents (> 10 years), we used the adolescent self-report as the referent [4, 8–10]. Differences in parent- and adolescent-reported MVPA (h∙wk.^− 1^) were determined by subtracting parent-reported MVPA from adolescent-reported MVPA (h∙wk.^− 1^).

### Statistical analyses

The Winsorization method was applied at the 99.5th percentile to minimize the impact of extreme values that were not plausible estimates of weekly MVPA [38]. Extreme values were recoded to the 99.5th percentile value within the respective distributions of parent- and adolescent- reported MVPA.

Sociodemographic characteristics were summarized using descriptive statistics, including measures of central tendency and variability for continuous variables and frequency and proportions for categorical variables. To assess the correlation between parent- and adolescent- reported MVPA, we computed weighted intraclass correlation coefficients (ICCs), which assesses correlation while accounting for within-dyad differences undetected by the Pearson’s correlation method. Paired t-tests were then used to determine differences between parent- and adolescent- reported MVPA [39]. Using established ICC cutoff values, we considered poor, fair, moderate, good, and very good agreement to have an ICC less than 0.2, between 0.2 and 0.4, between 0.4 and 0.6, between 0.6 and 0.8, and between 0.8 and 1, respectively [40].

Unadjusted parent-reports of MVPA, adolescent-reports of MVPA, and differences between parent- and adolescent- reports (h∙wk.^− 1^) were further summarized by sex, race/ethnicity, parent education, and household income. *P*-values were assessed using Wilcoxon Rank Sum or Kruskal-Wallis tests to determine if parent- and adolescent- reported median MVPA differed within groups of each sociodemographic characteristics. Data was weighted using propensity weights from the ABCD Study to approximate the American Community Survey by the U.S. Census [41].

To supplement ICC analyses, the Bland-Altman method was used to visualize agreement in parent- and adolescent- reported MVPA (h∙wk.^− 1^) by plotting parent-adolescent difference scores (vertical axis) by the median MVPA values of each parent-adolescent dyad (horizontal axis) [42]. Adolescent- reported MVPA was chosen as the criterion variable, given prior research suggesting that this age group is cognitively able to provide consistent survey responses [27]. Since we are not assuming a normal distribution of parent-adolescent difference scores, limits of agreement (LOA) were estimated using the 2.5th and 97.5th percentile values of the distribution of parent-adolescent difference scores. The average bias was estimated by the median value of the distribution of parent-adolescent difference scores [43]. Data analysis was performed using Stata 15.1 and RStudio v 1.3.1093.

## Results

In this sample, adolescents were aged 10–14 years old (mean 12.5 ± 0.9). Other sample sociodemographic characteristics were as follows: 50.0% were female; 38.9% were of racial/ethnic minority background; 10.0% had a parent with a high school education or less; 33.2% were in households earning less than $75,000 per year (Table 1).

**Table 1:** Sociodemographic characteristics of 4481 participants in the Adolescent Brain Cognitive Development (ABCD) Study, May 2020

Sociodemographic characteristics	Percent
**Adolescent characteristics**	
Sex	
Female	50.0%
Male	50.0%
Race/ethnicity	
White	61.1%
Latino / Hispanic	14.7%
Black	12.7%
Asian	7.8%
Native American	2.8%
Other	1.0%
**Parent characteristics**	
Education	
College education or more	90.0%
High school education or less	10.0%
Household income	
Less than $25,000	8.4%
$25,000 through $49,999	11.3%
$50,000 through $74,999	13.5%
$75,000 through $99,999	18.6%
$100,000 through $199,999	35.4%
$200,000 and greater	12.9%

Propensity weights from the Adolescent Brain Cognitive Development Study were applied based on the American Community Survey from the US Census

Table 2 presents adolescent- reported MVPA, parent-reported MVPA, and parent-adolescent absolute difference scores by hours per day (h·d^− 1^), days per week (d·wk.^− 1^), and hours per week (h∙wk.^− 1^). Adolescents reported engaging in a median of 2.17 [p25, p75 (0.00, 6.00)] hours of MVPA per week compared to 1.52 (0.29, 4.75) hours reported by their parents. Significant dyadic differences were found among each measure of MVPA, with absolute mean differences in parent- and adolescent- reports of 1.15 h·d^− 1^ (95% CI 1.08, 1.21), 1.68 d·wk.^− 1^ (95% CI 1.63, 1.72), and 4.89 h∙wk.^− 1^ (95% CI 4.64, 5.15). Within-dyadic MVPA reports from parents and adolescents, correlations were poor to moderate among each duration assessment of MVPA (all *p*-values were < 0.001), with ICCs ranging from 0.19 to 0.48.

**Table 2:** Parent- vs. adolescent- reported moderate-to-vigorous intensity physical activity (MVPA), ABCD Study, May 2020 (*N* = 4481)

	Adolescent	Parent	Difference	t	p	ICC (95% CI)
	Median (p25, p75)	Median (p25, p75)	Mean (95% CI)			
Hours/day	1.00 (0.00, 1.67)	0.50 (0.19, 1.07)	1.15 (1.08, 1.21)	34.85	**< 0.001**	0.19 (0.15, 0.23)
Days/week	2.00 (1.00, 4.00)	3.00 (2.00, 5.00)	1.68 (1.63, 1.72)	72.08	**< 0.001**	0.48 (0.45, 0.50)
Hours/week	2.17 (0.00, 6.00)	1.52 (0.29, 4.75)	4.89 (4.64, 5.15)	37.11	**< 0.001**	0.28 (0.24, 0.32)

**Fig. 1:**
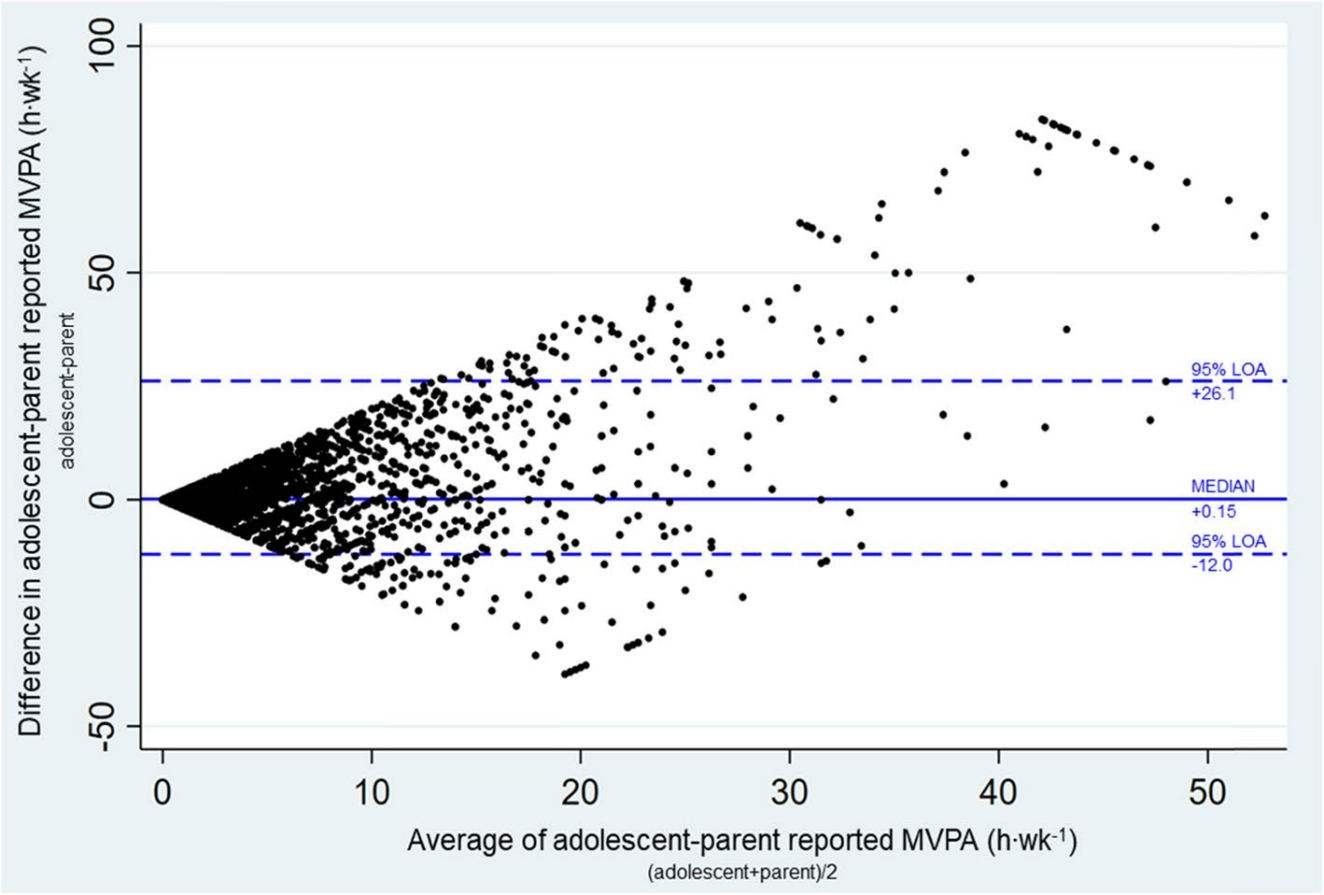
Bland-Altman Plot for agreement between adolescent-parent moderate-to-vigorous intensity physical (MVPA) activity reports. LOA = limits of agreement

A Bland-Altman plot (Fig. 1) further illustrated levels of within-dyad agreement between adolescent and parent-reported MVPA (h∙wk.^− 1^), revealing that the level of agreement between individual parent-adolescent dyads varied with a range of difference scores between − 38.5 to 83.86 h per week. Difference scores were located closer to the line of perfect agreement when average reported MVPA within dyads was closer to 0 h per week. Based on the calculated lower and upper bounds of agreement, 95% percent of differences in MVPA reports were expected to lie between − 12.0 and 26.1 h per week. Therefore, overall agreement was found with only 241 of 4841 (4.8%) of points lying outside the 95% interval. Sensitivity analyses stratified by sex, race, and income are shown in the Supplemental Appendix.

Table 3 summarizes parent-adolescent MVPA difference scores by sociodemographic characteristics. Significant differences in parent-adolescent MVPA by sociodemographic characteristics were found for parent-adolescent dyads by household income, with high income households having higher differences in parent-adolescent reports (difference score = 0.36 [p25, p75 (− 1.19, 3.04 h∙wk.^− 1^)]) compared to low-income households (difference score = 0.00 [p25, p75 (–1.15, 2.42 h∙wk.^− 1^)]). No significant differences in median MVPA hours per week within sex, race/ethnicity, or parent education level were found in this sample.

**Table 3:** Summary of difference scores in parent- and adolescent- reported moderate-to-vigorous intensity physical activity (MVPA) during the COVID-19 pandemic by sociodemographic characteristics in the Adolescent Brain Cognitive Development (ABCD) Study, May 2020, (N = 4481)

	Hours of MVPA/week			
	Adolescent reported	Parent reported	Difference score	p
Sociodemographic characteristics	Median (p25, p75)	Median (p25, p75)	Median (p25, p75)	
**Adolescent characteristics**				
Sex				0.09 ^a^
Female	2.01 (0.00, 6.00)	1.29 (0.28, 4.15)	0.19 (−0.86, 2.67)	
Male	2.33 (0.00, 6.65)	1.92 (0.42, 5.35)	0.00 (−1.29, 3.00)	
Race/ethnicity				0.05 ^b^
White	2.68 (0.50, 6.99)	1.92 (0.58, 5.35)	0.21 (−1.28, 2.96)	
Latino / Hispanic	1.50 (0.00, 5.01)	1.29 (0.17, 3.55)	0.00 (−0.96, 2.42)	
Black	2.00 (0.00, 6.00)	1.29 (0.14, 5.13)	0.00 (−1.05, 2.09)	
Asian	2.31 (0.66, 5.34)	1.52 (0.38, 3.55)	0.42 (−1.00, 2.71)	
Native American	1.50 (0.00, 4.00)	1.14 (0.14, 3.55)	−0.14 (−1.08, 1.92)	
Other	3.00 (0.00, 7.50)	0.66 (0.14, 3.44)	0.60 (−0.21, 4.74)	
**Parent characteristics**				
Highest parent education				1.00 ^a^
College education or more	2.33 (0.17, 6.00)	1.80 (0.38, 5.16)	0.09 (−1.19, 2.78)	
High school education or less	1.67 (0.00, 6.00)	1.14 (0.14, 3.55)	0.00 (−0.66, 3.00)	
Household income				**0.03** ^a^
$75,000 and greater	3.00 (0.66, 7.00)	1.92 (0.58, 5.35)	0.36 (−1.19, 3.04)	
Less than $75,000	2.00 (0.00, 5.01)	1.29 (0.17, 3.80)	0.00 (−1.15, 2.42)	

^a^ Wilcoxon Rank Sum test

^b^ Kruskal-Wallis test

## Discussion

To our knowledge, this is the first large, national study examining adolescent- and parent- reports of adolescent MVPA in the U.S. While parent and/or adult proxies are commonly used in younger children (aged < 10 years), the addition of the COVID RRR Survey to the ABCD study provided the novel opportunity to evaluate this methodological approach for use in adolescents during COVID-19 pandemic, when stay-at-home orders provided more opportunities for time with family members. We describe discrepancies between parent- and adolescent- reported physical activity, while also exploring reporting differences by several sociodemographic factors. We found that if an adolescent had no or little MVPA, the parent was generally accurate in reporting that their child was non-active. However, if the adolescent was active, particularly with very high levels of MVPA, there was less agreement between the parent's and youth's report. When examining sociodemographic factors associated with discrepancies in reported MVPA, there was a greater difference among MVPA parent and youth reporting among adolescents with higher MVPA, particularly in families with income > $75,000.

The finding that there was greater parent-adolescent agreement with lower levels of MVPA supports prior research results that indicate higher agreement on reporting of sports and outdoor activities among the lowest quartiles of reported activity [26]. In the context of the pandemic, non-active adolescents may be more likely to be home and engage in sedentary activities, such as recreational screen time, that can be better monitored by parents. It is also possible that there may be less activities to recall for both non-active adolescents and their parents, increasing the agreement of both reports (i.e., less measurement error). We initially hypothesized that there would be moderate to high parent-adolescent agreement in MVPA reporting during the pandemic for these reasons. Prior studies examining parent-adolescent agreement in physical activity among overlapping age groups reported Kappas of 0.11 (low) to 0.41 (fair). Our overall frequency agreement (ICC of 0.28) is within this range; however, the statistics were different (ICC vs Kappa) and the physical activity measure was slightly different (MVPA vs outside play, outdoor activities, leisure sports, organized sports, respectively) [12, 16, 26].

Conversely, if the adolescent is active, particularly with very high levels of MVPA, there is less agreement between the parent and the adolescent report, similar to previous findings [26]. On the adolescent side, this may be due to cognitive processes related to memory that make it harder to recall physical activity occurrence, frequency, and duration based on the structure of survey questions [44]. On the parent side, this may be due to the parent not knowing what their child is doing, particularly when the child is doing activities outdoors or overtraining. Active adolescents may be doing activities outside of the home with peers, teams, or individually of which parents may not be aware. For example, despite COVID-19 restrictions, some sports teams have maintained outdoor or distanced training routines. Moreover, at high levels of training for sports teams or competition, adolescents may overtrain [45] and may not want their parents to know how much they are training. Similarly, with eating disorders being exacerbated during the COVID-19 pandemic [46], more young people may be at engaging in excess covert exercise as a way to cope [47].

Overall, we found that adolescents reported more MVPA (h∙wk.^− 1^) than parents, similar to previous findings [16]. Adolescents may overestimate their intensity and time spent on MVPA [7]. Our findings also demonstrated greater discrepancies in subgroups with higher MVPA levels, such as adolescents from households with higher income. During the pandemic, adolescents from low-income neighborhoods may have been more affected by restricted access to safe outdoors spaces for MVPA [48], leading to lower MVPA levels and less parent-adolescent reporting discrepancies. Conversely, adolescents attending private schools were more likely to have in-person schooling during the pandemic [49] which could have allowed for more MVPA options and a greater parent-adolescent reporting discrepancy due to time away. One prior study of 9–12-year-olds similarly found that children of high socioeconomic status were more likely to report a higher frequency of outside play than their parents compared to children of low socioeconomic status [12]. Although we initially hypothesized that parent monitoring could have been improved due to more time at home from stay-at-home orders [31, 32], parents who are working from home may not have the flexibility to monitor their children while performing work duties and parents who are essential workers continue to go to in person work.

### Strengths and limitations

Strengths of our study stem from the utilization of a large, national adolescent study population with parent-adolescent dyads during the COVID-19 pandemic. Moreover, estimates of the adolescent’s MVPA were uniquely obtained from both the adolescent and the parent. However, despite these strengths, there are several limitations to our findings. Our data relies on reported estimates of adolescent MVPA through self-reports and parent reports, which may be subject to social desirability and recall bias. Unfortunately, more objective measures, including direct observation (criterion) or accelerometry were not collected to assess the potential bias of self-reported MVPA estimates, and in the absence of an objective measure, we cannot evaluate whether child- or parent- reports are more accurate. Moreover, there was some loss-to-follow-up and non-responders to the COVID-19 RRR survey administered by the ABCD study, meaning that there is risk for selection bias and limitations on the generalizability of these results. Non-responders were more likely to be from lower socioeconomic status backgrounds and racial/ethnic minorities, sociodemographic groups with lower average levels of MVPA. Additionally, the cross-sectional nature of this study limits our ability to establish causality, and may not be representative of longitudinal trends in MVPA agreement during the COVID-19 pandemic.

### Implications and conclusions

While most population-based studies of adolescents rely on self-report given their cognitive development [50], the addition of a parent proxy report in the ABCD COVID-19 RRR survey provided us the unique opportunity to examine the overall agreement between adolescent- and parent- reports of MVPA during the COVID-19 pandemic. However, we found that discrepancies in parent and adolescent agreement of MVPA persisted even during this time of closer cohabitation. Given the general decline in MVPA among adolescents and adults during the pandemic, family activities involving MVPA could both increase MVPA levels and improve agreement and monitoring of MVPA by parents. Schools and athletics programs can also involve parents more in the planning and accommodation of physical education classes or sports teams during the pandemic.

Our findings have implications for clinical and research practices in regards to measuring adolescent physical activity. Given decreases in MVPA during the pandemic [51] and discrepancies in parent-adolescent reporting of MVPA, parents could discuss and encourage MVPA with their adolescents. Pediatricians could consider assessing for and promoting MVPA for adolescents and their parents at primary care visits during the pandemic. These discrepancies also point to the need for research examining parent- and adolescent- reports of MVPA within the context of device-based measures (e.g., accelerometers). This information could be used to refine measures of adolescent MVPA and inform clinical practice given limited time in clinical visits (e.g., focusing on asking the adolescent only about MVPA if their report is more accurate than their parents’ report). Further studies could determine reasons for differences in parent-adolescent reporting of MVPA and use this knowledge to inform interventions for improved parental involvement and monitoring of MVPA.

## Supplementary Information

**Additional file 1: Supplemental Appendix**. **Figure S1**. Agreement between adolescent-parent MVPA reports among female adolescents. **Figure S2**. Agreement between adolescent-parent MVPA reports among male adolescents. **Figure S3**. Agreement between adolescent-parent MVPA reports among non-white adolescents. **Figure S4**. Agreement between adolescent-parent MVPA reports among white adolescents. **Figure S5**. Agreement between adolescent-parent MVPA reports in households with income $75,000+. **Figure S6**. Agreement between adolescent-parent MVPA reports in households with income <$75,000+.

## Data Availability

Data used in the preparation of this article were obtained from the ABCD Study (https://abcdstudy.org), held in the NIMH Data Archive (NDA). Investigators can apply for data access through the NDA (https://nda.nih.gov/).
