# Supplementary material for: Parent-adolescent agreement in reported moderate-to-vigorous intensity physical activity during the COVID-19 pandemic
Source: BMC Public Health. 2022 Feb 16;22:332. doi: 10.1186/s12889-022-12530-4 (PMC8851835; doi:10.1186/s12889-022-12530-4)
Supplement: Supplementary file 1 — Additional file 1: Supplemental Appendix. Figure S1. Agreement between adolescent-parent MVPA reports among female adolescents. Figure S2. Agreement between adolescent-parent MVPA reports among male adolescents. Figure S3. Agreement between adolescent-parent MVPA reports among non-white adolescents. Figure S4. Agreement between adolescent-parent MVPA reports among white adolescents. Figure S5. Agreement between adolescent-parent MVPA reports in households with income $75,000+. Figure S6. Agreement between adolescent-parent MVPA reports in households with income <$75,000+. [file 12889_2022_12530_MOESM1_ESM.docx]

**Supplemental Appendix**

**Measures from COVID Rapid Response Research Survey 1**

ABCD Physical Activity questionnaires were adapted from the Youth Risk Behavior Survey (YRBS) [1, 2] and the International Physical Activity Questionnaire (IPAQ) Short-form [3, 4].

**Adolescent Questions**

Question:

During the past week, on how many days did you do moderate or vigorous physical activities like heavy lifting, running, aerobics, or bicycling? (Moderate or vigorous physical activities refer to activities that take moderate to hard physical effort and make you breathe harder than normal)

Responses:

0 = No moderate or vigorous physical activities; 1 = 1 Days; 2 = 2 Days; 3 = 3 Days; 4 = 4 Days; 5 = 5 Days; 6 = 6 Days; 7 = 7 Days; 8 = Don't know.

Question:

How much time did you usually spend doing moderate or vigorous physical activities on one of those days?

Responses:

Minutes: 0 = 0 minutes; 1 = 10 minutes; 2 = 20 minutes; 3 = 30 minutes; 4 = 40 minutes

Hours: 0 = 0 hours; 1 = 1 hour; 2 = 2 hours; 3 = 3 hours; 4 = 4 hours; 5 = 5 hours; 6 = 6 hours; 7 = 7 hours; 8 = 8 hours; 9 = 9 hours; 10 = 10 hours; 11 = 11 hours; 12 = 12 hours; 13 = 13 hours; 14 = 14 hours; 15 = 15 hours; 16 = 16 hours; 17 = 17 hours; 18 = 18 hours; 19 = 19 hours; 20 = 20 hours; 21 = 21 hours; 22 = 22 hours; 23 = 23 hours

MVPA was calculated as a frequency of MVPA x time spent doing MVPA= estimated MVPA h∙wk^-1^ during COVID-19.

**Parent Questions**

Question:

During the past week, on how many days per week did your child do moderate or vigorous physical activities like heavy lifting running aerobics or bicycling?

Responses:

0 = No moderate or vigorous physical activities; 1 = 1 Days; 2 = 2 Days; 3 = 3 Days; 4 = 4 Days; 5 = 5 Days; 6 = 6 Days; 7 = 7 Days; 8 = Don't know.

Question:

How much time did your child usually spend doing moderate or vigorous physical activities on one of those days?

Responses:

Minutes: 0 = 0 minutes; 1 = 10 minutes; 2 = 20 minutes; 3 = 30 minutes; 4 = 40 minutes

Hours: 0 = 0 hours; 1 = 1 hour; 2 = 2 hours; 3 = 3 hours; 4 = 4 hours; 5 = 5 hours; 6 = 6 hours; 7 = 7 hours; 8 = 8 hours; 9 = 9 hours; 10 = 10 hours; 11 = 11 hours; 12 = 12 hours; 13 = 13 hours; 14 = 14 hours; 15 = 15 hours; 16 = 16 hours; 17 = 17 hours; 18 = 18 hours; 19 = 19 hours; 20 = 20 hours; 21 = 21 hours; 22 = 22 hours; 23 = 23 hours

MVPA was calculated as a frequency of MVPA x time spent doing MVPA= estimated MVPA h∙wk^-1^ during COVID-19.

**Supplemental Figures**

Figure S1. Agreement between adolescent-parent MVPA reports among female adolescents


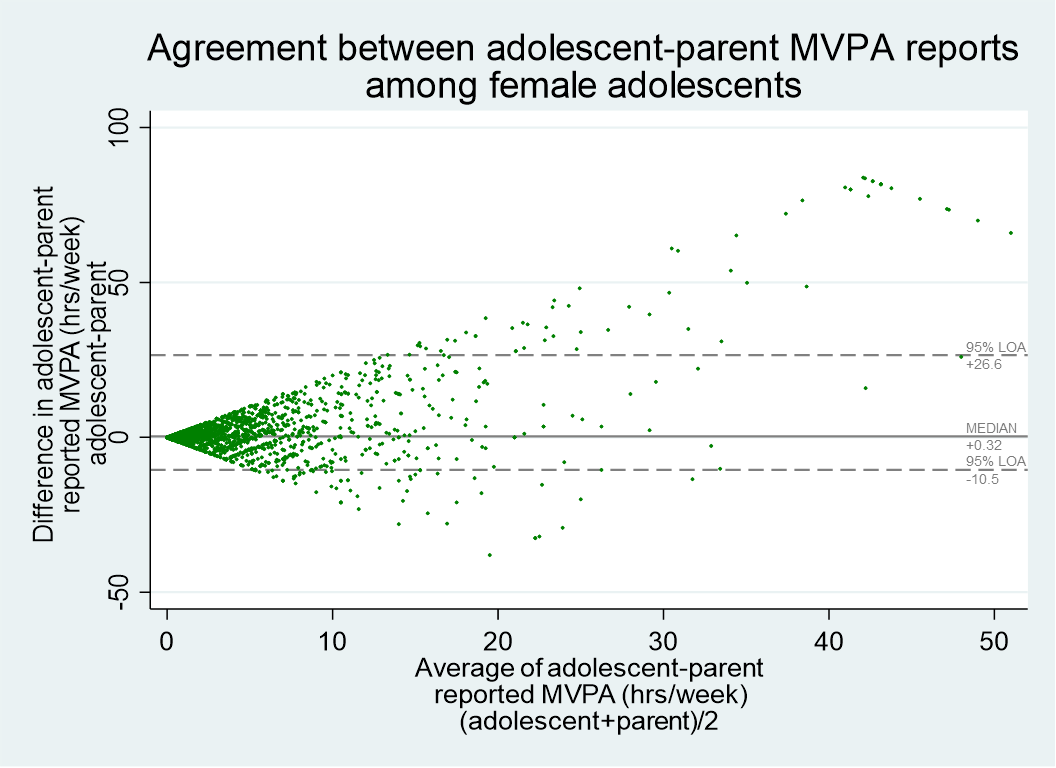


LOA = limits of agreement

Figure S2. Agreement between adolescent-parent MVPA reports among male adolescents


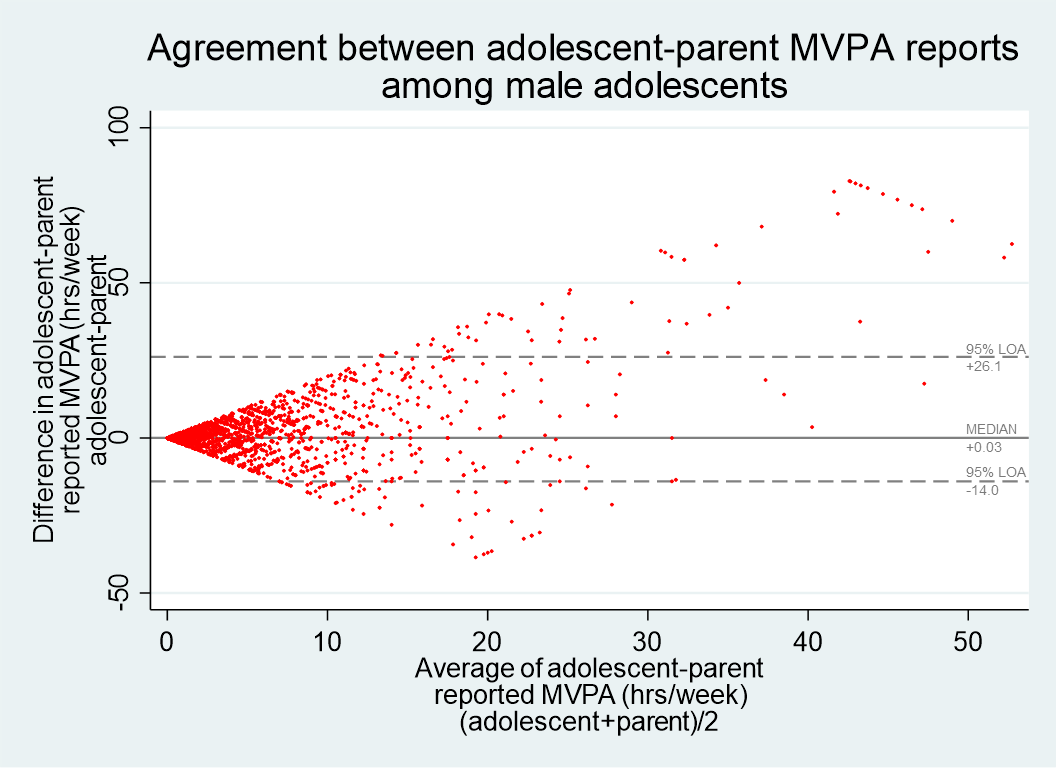


LOA = limits of agreement

Figure S3. Agreement between adolescent-parent MVPA reports among non-white adolescents


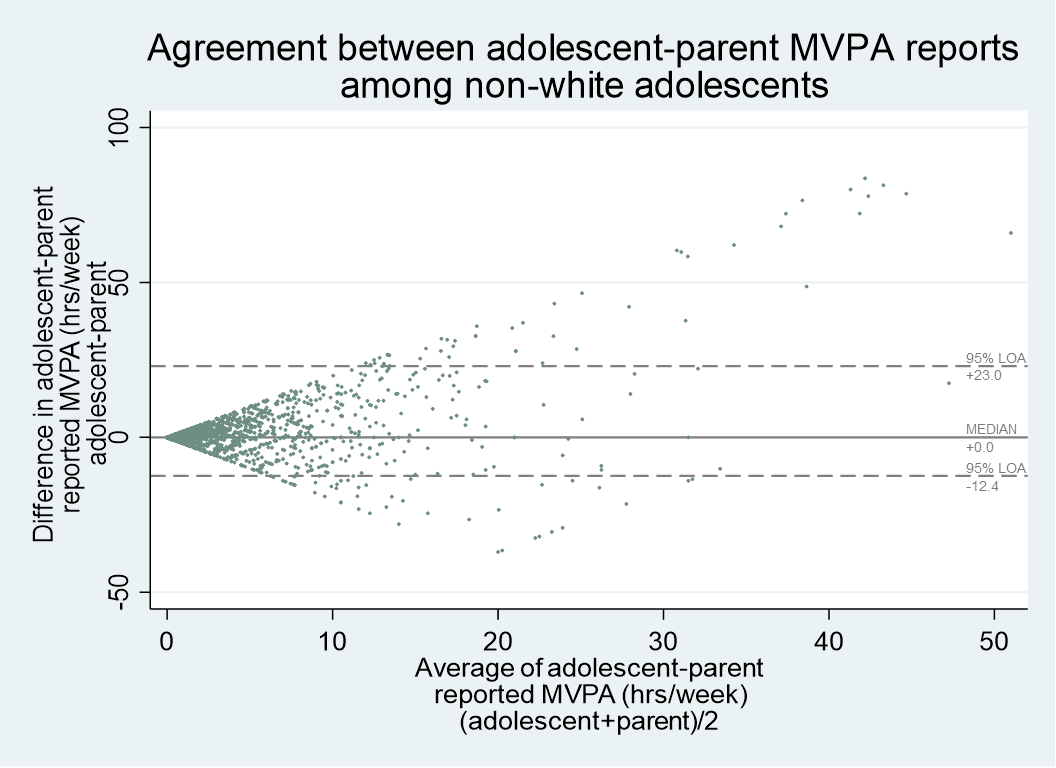


LOA = limits of agreement

Figure S4. Agreement between adolescent-parent MVPA reports among white adolescents


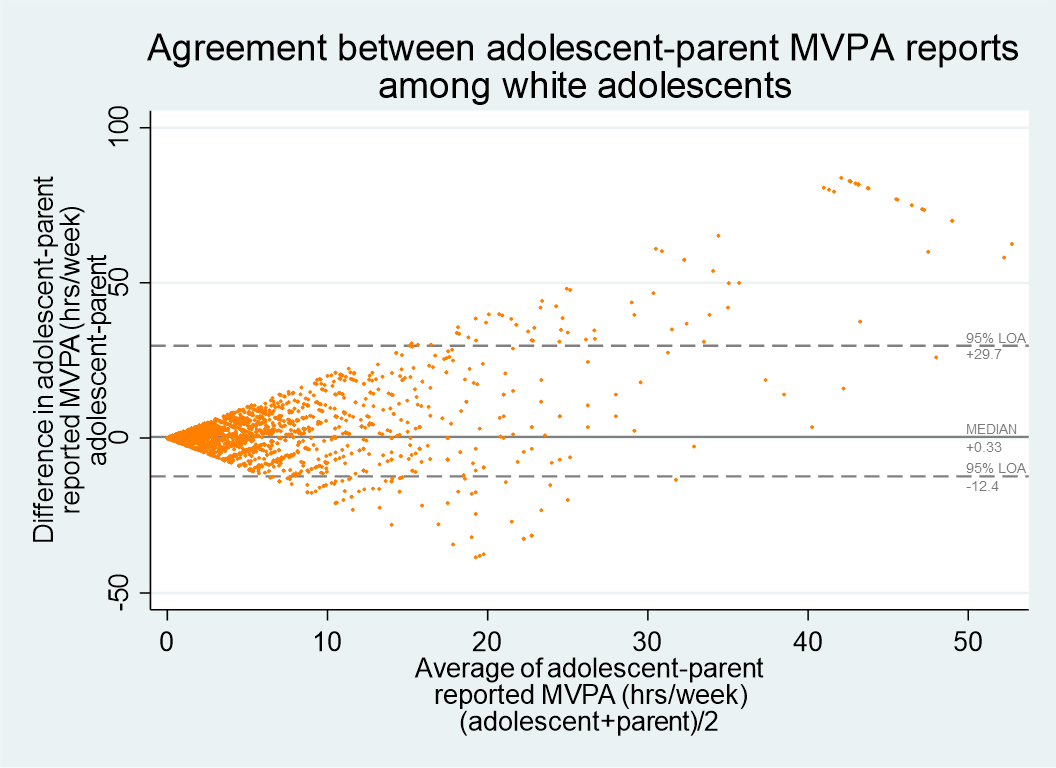


LOA = limits of agreement

Figure S5. Agreement between adolescent-parent MVPA reports in households with income $75,000+


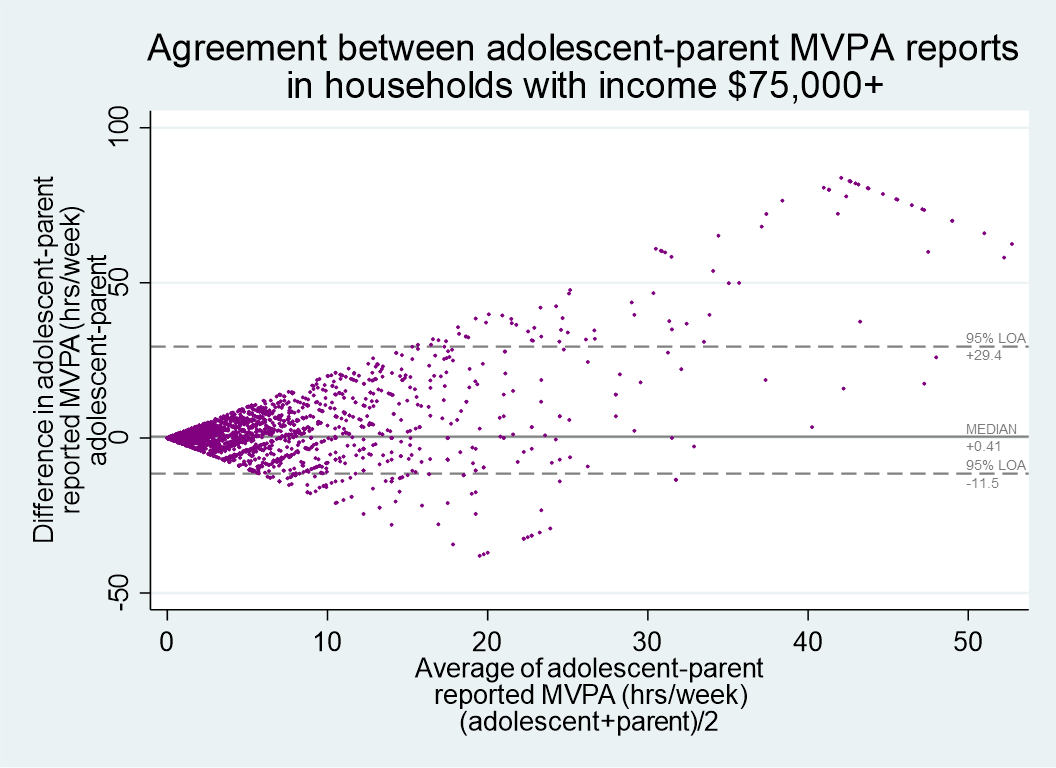


LOA = limits of agreement

Figure S6. Agreement between adolescent-parent MVPA reports in households with income <$75,000


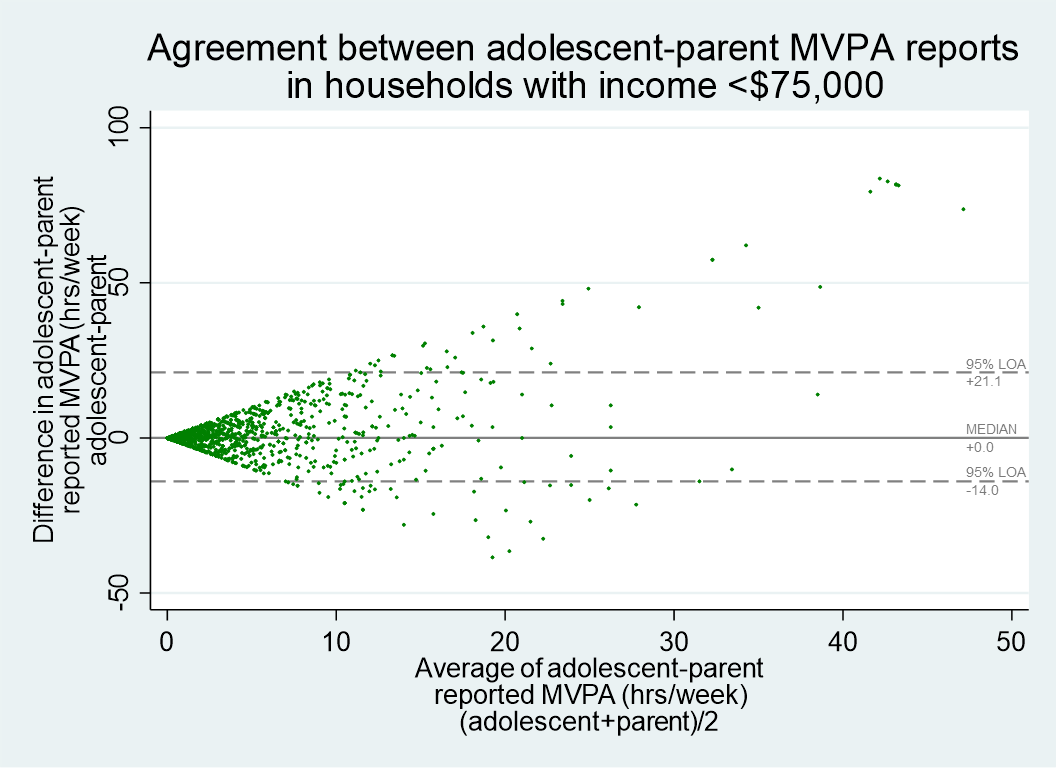


LOA = limits of agreement
